# Oral health issues of the disabled population: a growing concern in China

**DOI:** 10.3389/fpubh.2025.1585300

**Published:** 2025-05-20

**Authors:** Xing Wang

**Affiliations:** China Rehabilitation Research Center, Capital Medical University, Beijing, China

**Keywords:** oral health, disability, policy and disabilities, dental, health and nutrition education

## Introduction

Oral health is a critical yet often overlooked aspect of overall wellbeing, particularly for vulnerable populations such as individuals with disabilities (1). In China, where the disabled population exceeds 85 million, oral health disparities are increasingly recognized as a pressing public health issue (2). Despite progressive policies like the Healthy China 2030 plan, which emphasizes improved medical services for disabled individuals, significant gaps remain in addressing their oral health needs (3). This commentary argues that China must prioritize the development of specialized dental services and leverage global best practices to create a more inclusive and equitable oral healthcare system for its disabled population.

## The burden of oral health disparities among the disabled

Disabled individuals in China face disproportionately high rates of oral diseases, including dental caries and periodontal diseases, due to a combination of physical, financial, and systemic barriers. According to the World Health Organization (WHO), people with disabilities are twice as likely to develop oral health issues compared to the general population (4). Nationwide, delayed care due to financial or logistical challenges leads to higher caries and periodontal disease rates among disabled populations. For instance, in Guangdong, intellectually disabled adolescents (12–17 years) exhibited a caries prevalence of 53.5% and a DMFT index of 1.5 ± 2.0, exceeding the national averages of 38.5% and 0.86, respectively (5). In economically disadvantaged regions, treatment inequities are starker, with near-zero filling rates for caries among disabled individuals (6). Periodontal health is equally concerning. A large-scale study of disabled children and adolescents (0–18 years) reported progressive increases in gingival bleeding (28.36%) and calculus deposition (42.14%) with age (7). Health outcomes also vary by disability type: visually impaired individuals show higher caries prevalence (78.64%), calculus accumulation (67.96%), and malocclusion rates (49.51%) (8). Despite limited data, these findings underscore urgent systemic gaps in China's oral healthcare infrastructure, necessitating equitable, disability-inclusive services. The above-mentioned circumstances could lead to more profound consequences, as poor oral health is closely linked to systemic conditions like cardiovascular disease and diabetes, which are prevalent among disabled individuals (9). Regrettably, existing surveys lack comprehensive national data, particularly on the oral health status of adults with disabilities. This gap may stem from limited geographic coverage, challenges in data collection, systemic underprioritisation of oral health in disability care, and insufficient dedicated funding. These challenges are compounded by the diverse nature of disabilities, which require tailored approaches to oral healthcare (7, 10). For example:

- Physical and visual impairments often hinder effective oral hygiene practices, leading to a higher prevalence of dental problems.
- Hearing impairments create communication barriers that delay diagnosis and treatment.
- Intellectual and developmental disabilities, such as autism, may result in behavioral challenges that complicate standard dental procedures.

## Policy progress and persistent gaps

China has made significant strides in improving healthcare access for disabled individuals through progressive policies and legal frameworks. The Healthy China 2030 plan, for instance, calls for enhanced barrier-free facilities in medical institutions and upgraded services for disabled patients (3). Additionally, the establishment of the first regional Dental Care Fund for Disabled Persons in 2020 represents a step forward in addressing oral health disparities (11). However, these efforts fall short of meeting the growing demand for specialized dental services. China's healthcare system, which includes over 30,000 public hospitals and 130,000 private dental clinics, lacks sufficient professionals and facilities equipped to serve disabled patients (12). To ameliorate this situation, the China Rehabilitation Research Center, as the sole tertiary comprehensive hospital directly under the China Disabled Persons' Federation (CDPF), has expanded its stomatology department to accommodate more disabled patients and provide them with professional oral healthcare services. However, these initiatives remain insufficient to address the scale of the problem.

Recent national data reveal a severe shortage of dental professionals in China, with a dentist-to-population ratio of 1:7,768—far below the WHO-recommended standard of 1:5,000 (13). Compounding this issue, a WHO cross-sectional study in developing countries found that over 60% of Chinese adults face unmet oral healthcare needs due to socioeconomic inequities (14). For individuals with physical or intellectual disabilities, access barriers are even more pronounced. In Hong Kong, 70% of patients with special needs did not visit a dentist within 3 years despite experiencing tooth pain (15). More concerningly, a recent survey analysis revealed that while parents of children with disabilities demonstrated improved oral health awareness—with 78.98% expressing strong proactive attitudes toward maintaining their children's oral health−49.75% of these children had never visited a dental care facility (16).

## Addressing oral health disparities among the disabled population in China

To bridge these gaps, China can draw inspiration from global best practices. For example:

- The United Kingdom has established specialized dental care centers for disabled individuals, ensuring tailored services and improved access (17).
- Japan has implemented nationwide training programs for dental professionals to better serve disabled patients (18).
- The International Association for Disability and Oral Health (IADH) has developed e-learning modules to enhance oral healthcare for disabled individuals, leveraging technology to improve training and service delivery (19).

These examples highlight the potential of innovative approaches, such as artificial intelligence (AI) and tele-dentistry, to address oral health disparities. China's current policies promoting technology for assisting disabled individuals align well with these strategies, offering a unique opportunity to integrate advanced solutions into its healthcare system (20).

Within China's evolving healthcare system, developing targeted strategies to address oral health inequities among disabled individuals requires a multi-dimensional approach that combines policy innovation with community-based solutions. Policy-level interventions must form the foundation of these efforts, beginning with the integration of essential dental services—including comprehensive screenings, fluoride applications, and basic restorative treatments—into the national primary healthcare package (3). This integration should be supported by dedicated fiscal allocations and an enhanced medical insurance reimbursement scheme, potentially increasing coverage for disabled patients from the current average of 60 to 80% for common procedures.

To overcome physical accessibility barriers, a three-tiered infrastructure improvement strategy should be implemented. First, establishing a collaborative network between local disability associations and community health centers to create standardized, barrier-free dental clinics equipped with wheelchair-accessible operatories and adaptive equipment. Second, expanding the successful mobile dental unit model pioneered in Guangdong-Hong Kong, with particular focus on underserved rural communities in central and western regions (5, 15). Third, implementing mandatory disability-competency training for oral health professionals, covering communication strategies for patients with various disabilities.

The current service delivery gap remains particularly concerning in primary care settings. Despite national targets of 70% coverage, recent data indicates only 30% of disabled individuals are enrolled in family physician programs (21). To address this, oral health assessments should be incorporated as mandatory components of the disabled population's family doctor service package, with quality indicators tied to performance evaluations (22). Additionally, innovative solutions like teledentistry consultations could bridge geographical barriers, while community health workers could be trained to provide basic oral health education and preventive care.

## Conclusion

The oral health needs of China's disabled population represent a critical yet underaddressed public health challenge. While progressive policies and initiatives have laid a foundation for improvement, significant gaps in service delivery and accessibility persist. By learning from global best practices and leveraging innovative technologies, China can develop more inclusive and effective oral healthcare solutions for its disabled population. Addressing these disparities is not only a matter of health equity but also a crucial step toward achieving the broader goals of the *Healthy China 2030 plan*. Continued collaboration among governments, healthcare providers, and advocacy groups will be essential to ensure that these efforts reach their full potential and create a more inclusive healthcare system for all.
